# Approaches to modelling the shape of nanocrystals

**DOI:** 10.1186/s40580-021-00275-6

**Published:** 2021-09-09

**Authors:** Christina Boukouvala, Joshua Daniel, Emilie Ringe

**Affiliations:** 1 Department of Materials Science and Metallurgy, University of Cambridge, Cambridge, CB3 0FS UK; 2 Department of Earth Sciences, University of Cambridge, Cambridge, CB2 3EQ UK

**Keywords:** Nanoparticle shape, Wulff construction, Winterbottom construction, Shape modelling, Shape modelling tools

## Abstract

Unlike in the bulk, at the nanoscale shape dictates properties. The imperative to understand and predict nanocrystal shape led to the development, over several decades, of a large number of mathematical models and, later, their software implementations. In this review, the various mathematical approaches used to model crystal shapes are first overviewed, from the century-old Wulff construction to the year-old (2020) approach to describe supported twinned nanocrystals, together with a discussion and disambiguation of the terminology. Then, the multitude of published software implementations of these Wulff-based shape models are described in detail, describing their technical aspects, advantages and limitations. Finally, a discussion of the scientific applications of shape models to either predict shape or use shape to deduce thermodynamic and/or kinetic parameters is offered, followed by a conclusion. This review provides a guide for scientists looking to model crystal shape in a field where ever-increasingly complex crystal shapes and compositions are required to fulfil the exciting promises of nanotechnology.

**Supplementary Information:**

The online version contains supplementary material available at 10.1186/s40580-021-00275-6.

## Introduction

Nanocrystals, defined as crystalline particles of size ranging from 1 to 1000 nm, have found a myriad of applications across science and engineering, for instance in optical devices [1, 2], chemical catalysis [3, 4], drug delivery [5–7], and biological sensors [8–10], to name a few. Unlike in the bulk, the size and shape of nanocrystals has a profound influence on their properties, driving interest and effort in precise control strategies for both top-down fabrication and bottom-up synthesis approaches. In bottom-up approaches, thermodynamic and kinetic mechanisms dictate atomic assembly. Understanding such effects, possible through the various crystal growth models developed over the last century, is key to rationalizing and predicting the shape of crystalline nanoparticles.

**Fig. 1:**
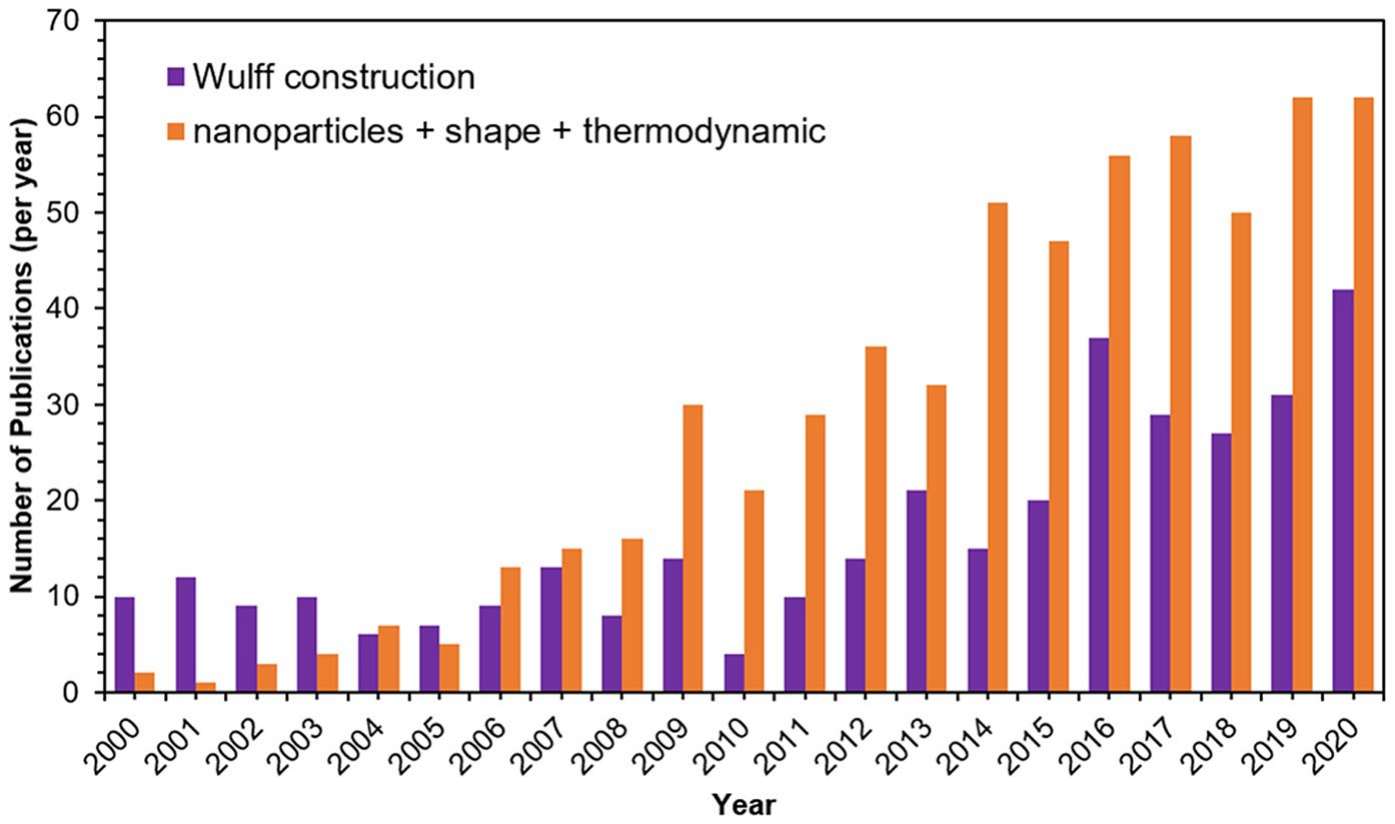
Yearly number of publications featuring the words “Wulff construction” (purple) or all of the words “nanoparticle”, “shape” and “thermodynamic” (orange) in their title or abstract. Data obtained from https://app.dimensions.ai/discover/publication

Interest in nanocrystal shape and its control has, therefore, followed the trend of interest in nanostructures. Marks and Peng [11] showed in 2016 that the number of publications using “nanoparticle” as a keyword increased approximately linearly with time; this trend has continued through 2020 (Additional file 1: Fig. S1). However, only a small fraction of these 10,000s of papers are relevant to nanoparticle shape research. Figure 1 shows the much smaller number, but similar steady increase, of papers including in their title or abstract the words “nanoparticle”, “shape” and “thermodynamic”, or the words “Wulff construction”. This steady rise in interest in Wulff construction and nanoparticle shape is poised to continue as scientists explore unusual crystal systems and ever-increasing nanoparticle complexity.

The thermodynamic equilibrium shape of a nanocrystal, the simplest one to model, is governed by surface energy minimisation, as determined by Gibbs in 1873 [12]. This was formalized in 1901 by Wulff [13] into the “Wulff construction,” sometimes referred to as the “classic” [14] or “thermodynamic” [15] Wulff construction. According to this approach, the equilibrium shape of single crystals is determined from a gamma plot, i.e. a plot of the orientation-dependent surface free energy (surface energy for simplicity subsequently). Extensions to this purely thermodynamic, single crystal Wulff construction have been developed over the past decades to incorporate twinning [16], alloys [17], substrate(s) [18–20], and kinetic effects [21] such that most shapes and conditions can now be modelled. These mathematical models are all general, and specific crystallographic implementation, e.g. for the common face-centered cubic (FCC) metals, often relies on software development for the visualization and quantification of shapes. The development of computer-based interfaces implementing shape models is much newer than the models themselves and buoyed by both the open-source movement and the wide availability of computing resources. Unlike the mathematically unique models, however, the gamut of usable coding languages and interfaces provides a plurality of valid ways to implement and distribute Wulff computational tools, each with advantages and limitations.

This short review starts with a brief overview and disambiguation of the various mathematical models and terminologies used to model crystal shapes, from the century-old Wulff construction to the year-old (2020) approach to describe supported twinned nanocrystals. Next, we explore the multitude of published software implementations of Wulff-based shape models, describing for each their technical aspects, advantages and limitations. Finally, a discussion of the scientific applications of shape models to either predict shape or use shape to deduce thermodynamic and/or kinetic parameters is offered, followed by a conclusion.

## Wulff and Wulff-related constructions

In this section, the classic thermodynamic Wulff construction and its mathematical basis will first be introduced, followed by an overview of derivative but mathematically distinct Wulff-related constructions for (nano)crystals under various constraints such as twinned crystals, crystals governed by kinetic growth, alloys and supported crystals. We aim to keep this brief and the reader is directed to several more detailed publications for further details, if needed [11, 21–23]. This section concludes with a discussion of the often-confusing terminology used to describe the Wulff construction and its variants.

### The thermodynamic Wulff construction

**Fig. 2:**
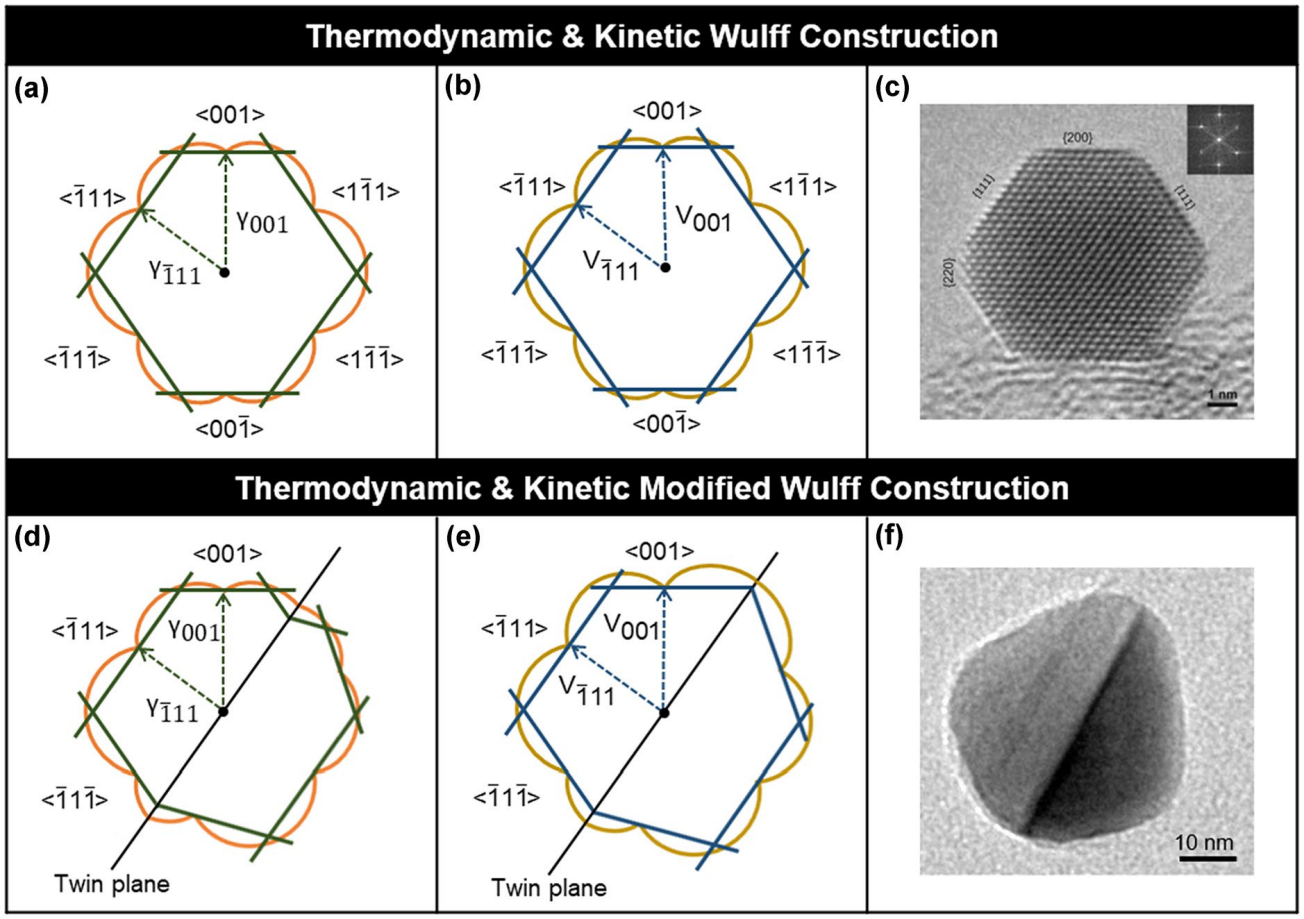
Single crystal and modified (twinned) Wulff constructions. Gamma and v-plots viewed along < 110 > for the (**a**) thermodynamic, (**b**) kinetic, (**d**) thermodynamic modified and (**e**) kinetic modified with re-entrant enhancement Wulff constructions. γ and v correspond to surface energy and growth velocity, respectively, and the twin energy/twin growth velocity is considered to be negligible. Examples of TEM images of (**c**) a single crystal AgI octahedron, reprinted from [25] with permission from Elsevier, and (**f**) a twinned Ag right bipyramid, adapted with permission from [26]. Copyright (2021) American Chemical Society

The “original” thermodynamic Wulff construction applies to the case of a single crystal in thermodynamic equilibrium. It states that the normal vector length to any external crystal facet will be proportional to the surface free energy of that facet, commonly expressed as:

1 $$\begin{array}{c}{\gamma }_{i}= h_{i}/\lambda,\end{array}$$

where *γ*_*i*_ is the orientation-dependent surface free energy of facet *i*, *h*_*i*_ is the normal distance from the centre of the particle (also referred to as the ‘Wulff point’ [24]) to the facet *i*, and λ is a constant accounting for volume. Graphically, this can be represented in a gamma plot, as shown in Fig. 2; the inner envelope of planes from the gamma plot represents the thermodynamic crystal shape. Equivalently, a vector definition of surface energy can be used to produce a set of points (*S*_*w*_) defining the crystal’s thermodynamic equilibrium shape:

2 $$\begin{array}{c}{S}_{w}=\left\{x:x.\widehat{n}\le\uplambda {\gamma }_{\left(\widehat{n}\right)}{\text{ for all unit vectors } }\widehat{ n}\right\}\end{array},$$

where $\widehat{n}$ is a unit vector defined by the crystallographic orientation of a facet (hkl) where ${\gamma }_{\left(\widehat{n}\right)}$ is the orientation-dependent surface energy vector and $\uplambda$ is as previously defined.

Wulff’s construction from 1901 was formally proven later by von Laue (1943) [27] and then generalised to curved surfaces by Dinghas (1944) [28]. It is worth noting that even though most of the analysis in Wulff’s 1901 paper is based on growth rate experiments (i.e. kinetics), Wulff assumed (not necessarily correctly) a direct relationship between surface free energies and growth rates, hence the construction is appropriate for thermodynamic conditions.

**Fig. 3:**
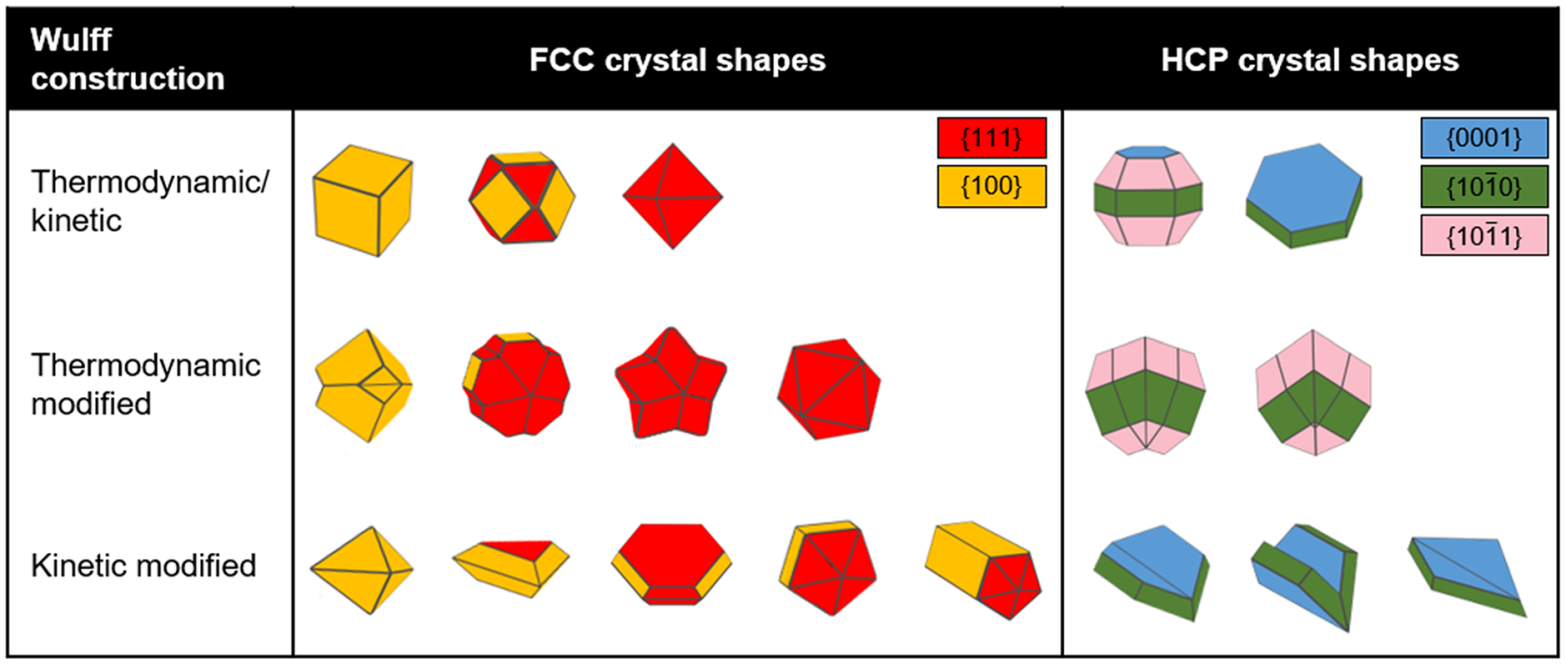
Nanocrystal shapes in the single crystal thermodynamic/kinetic, thermodynamic modified and kinetic modified Wulff constructions for FCC and HCP crystal structures. The FCC structures are twinned along the (111) plane, while the twinning plane varies for HCP as described in ref. [31] Facets are colour coded according to legend and shapes were created in Crystal Creator [32]

This thermodynamic approach, while limited to single crystals, has been abundantly used to understand and describe the shape of nanocrystals. In the common FCC system, adopted by Au, Ag, Cu, Al, and many others, thermodynamic crystal shapes lay somewhere in between a cube and an octahedron, *i.e.* a cuboctahedron (Fig. 3), because this shape exposes the close-packed {111} and the densely packed {100} facets, both of low surface energy [29, 30]. Meanwhile, single crystal of hexagonal close-packed (HCP) elements such as Mg, where the {0001} plane is close-packed, form hexagonal prisms and related structures as shown in Fig. 3.

### The thermodynamic modified (twinned) Wulff construction

The introduction of internal planar defects, namely twin boundaries formed at the nucleation and early stage of growth, leads to different underlying symmetry and potentially more complex crystal shapes. Particles with a single twin boundary or parallel sets of twin boundaries are referred to as singly-twinned or lamellar twinned particles (LTPs) [16], while those with more than one twin boundary are often called multiply twinned particles (MTPs) [16]. Twinned crystals can be regarded as linked single crystal subunits, joined along specific twin boundary(ies) and closed, for MTP, at the cost of lattice strain [11, 33]. For instance, the decahedral particles common in FCC systems are MTPs consisting of five tetrahedra each sharing a face and all sharing a common edge, while icosahedral particles are an assembly of 20 tetrahedra each sharing a face.

For twins, the thermodynamic equilibrium shape is found by determining the thermodynamic Wulff shape for each crystal subunit, taking into account the twin boundary energy(ies), then “assembling” the final structure from these subunits. This approach, proposed by Marks in 1983 [16], is called the thermodynamic modified Wulff construction and is expressed as the superset of Wulff shapes *S*_*m*_ of the individual single-crystal subunits:

3 $${S}_{m} =\left\{x : \left(x-{o}_{m}\right).\widehat{n} \le {\uplambda }_{\mathrm{m}}{\gamma }_{m\left(\widehat{n}\right) \,}{\text{for all unit vectors} }\,\widehat{n}\right\},$$

where ${o}_{m}$ are the origins for each subunit, ${\uplambda }_{\mathrm{m}}$ is the constant to account for the volume of each subunit and ${\gamma }_{m\left(\widehat{n}\right)}$ is the surface energy which includes the twin boundary energy of ${\alpha }_{mn}{\gamma }_{t}$ for each subunit ‘m’ adjacent to a subunit ‘n’ (where ${\gamma }_{t}$ is the twin boundary energy per unit area). Resulting additional constraints are:

4 $$\begin{array}{c}{\alpha }_{mn}+ {\alpha }_{nm}=1,\end{array}$$

5 $$\begin{array}{c}{S}_{mn}^{t}= {S}_{nm}^{t}\end{array},$$

where ${S}_{mn}^{t}$ is the bounding twin surface of subunit m to subunit n. Interestingly, whilst single crystals and subunits of a twinned structure require convex shapes, LTPs and MTPs can have concave, re-entrant surfaces which appear odd but do minimize total surface energy. For instance, the thermodynamic shape of a small FCC MTP, the Marks decahedron, exposes such grooves, as does a MTP with more stable {111} facets (Fig. 3). The thermodynamic shape of a singly twinned bipyramid exposing {100} facets also contains these features. Similarly, re-entrant corners are expected in some of the thermodynamic HCP shapes, as shown in Fig. 3. These are not always observed experimentally because of kinetic effects, as explained below.

### The kinetic and modified kinetic Wulff constructions

A nanocrystal shape will only reach thermodynamic equilibrium given sufficient time and energy; many syntheses yield non-equilibrium shapes owing to kinetic effects. The kinetic Wulff construction aims to include these effects; it was first formally used by Frank et al*.* in 1958 [34], but the significance of kinetics in crystal growth was already known [35]. For single crystals, the model’s difference from its thermodynamic equivalent is that growth velocities (${\nu }_{i}$) are used rather than surface free energies (${\gamma }_{i}$) to determine the crystal shape:

6 $$\begin{array}{c}{v}_{i}= h_{i(t)}/{\uplambda }_{(t)}\end{array},$$

where the facet distance from the centre (${h}_{i(t)})$ and the Wulff constant ${\uplambda }_{(t)}$ are now function of time. The kinetic Wulff shape can thus be determined with the growth velocity variant of the $\gamma$-plot called a $\nu$-plot (Fig. 2). Analogously to the thermodynamic construction, the shape is made of all points $x$ following:

7 $${S}_{K} =\left\{x : x.\widehat{n} \le {\lambda }_{\left(t\right)}{\nu }_{\left(\widehat{n}\right)} \,{\text{for all unit vectors }}\widehat{n}\right\},$$

where ${\nu }_{\left(\widehat{n}\right)}$ is the orientation-dependent growth velocity.

In the case of twinned crystals grown in kinetic conditions, a treatment similar to that of the thermodynamic approach can be applied by forming individual kinetically grown crystals (Eq. 6) and assembling them as previously (Eq. 3), assuming zero growth velocity for the twin plane(s) [21]. This yields the modified kinetic Wulff construction. However, additional kinetic effects are responsible for the shapes observed, such as preferential growth in concave facets and enhanced growth along twin boundaries [21]. These effects are well established [36] and hold true for twin boundaries, re-entrant surfaces and disclination lines.

The kinetic single crystal Wulff construction allows for shapes beyond the thermodynamic equilibrium by taking into account the growth-directing effects of surfactants, underpotential deposition, or other reaction additives and conditions [37–42]. For instance, Ag underpotential deposition on Au nanoparticles blocks the growth of low-index facets, allowing for rather exotic shapes such as {310}-bound truncated ditetragonal prisms and {720}-bound concave cubes, while poly(vinyl pyrrolidone) (PVP) directs the growth of Ag to preferentially form nanocubes with {100} facets rather than the thermodynamically expected cuboctahedra [43, 44].

The ability to add growth enhancements to re-entrant facets as well as disclinations and twin boundaries enabled the modified (twinned) Wulff construction to model a host of observed but previously unexplained nanocrystal shapes, i.e. shapes impossible to obtain by simply changing the surface energies/growth velocities (Fig. 3). These include sharp decahedra and bipyramids in FCC crystals [21], and sharp folded structures in HCP materials, to name a few [31].

### The alloy Wulff construction

The ability of homogeneous alloy particles to form more stable structures via surface segregation, and the resulting change in shape, has been incorporated in the alloy Wulff construction [17] and can be written as:

8 $$\begin{array}{c}{h}_{\left(i\right)}= \frac{{\gamma }_{\left(i, {C}_{1}^{S}, {C}_{1}^{V},{C}_{2}^{S},{C}_{2}^{V}\dots \right)}}{\left\{\Lambda - \Delta G\right\}},\end{array}$$

where ${C}_{n}^{S}$ and ${C}_{n}^{V}$ are the surface and volume fractional concentration of element n, respectively, and ${\gamma }_{\left(i, {C}_{1}^{S}, {C}_{1}^{V},{C}_{2}^{S},{C}_{2}^{V}\dots \right)}$ is the face- and composition-dependent surface energy. $\Lambda$ is a Lagrangian multiplier with which energy is minimized with respect to changes in bulk and surface segregation and $\Delta G$ is the difference between the free energy per unit volume of the final bulk composition after surface segregation and that of an initial composition where the surface and bulk concentrations are assumed to be equal. The lowering of overall surface energy thus competes with the resulting changes in bulk energy due to changes in bulk concentration. The resulting nanoparticle shape and surface composition is dictated by the composition-dependent bulk and surface energy curves as well as the particle size, an unusual feature for the typically size-independent Wulff approach. While experimental, systematic studies of this effect are scarce, several observations and predictions of size-dependent segregation have been reported and are summarized in ref [45].

### Constructions for supported nanocrystals

**Fig. 4:**
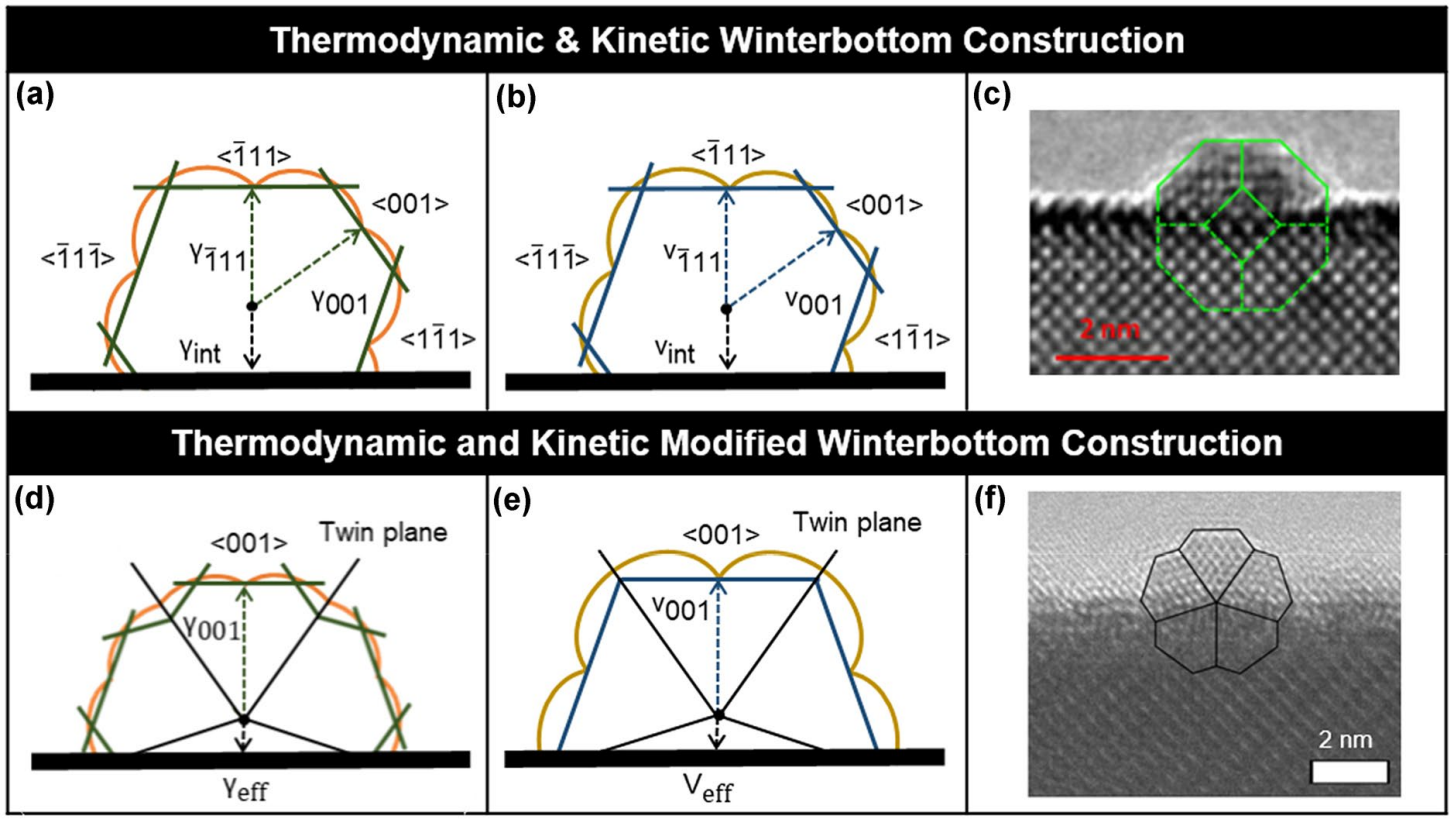
Winterbottom and related constructions for supported crystals. Gamma and v-plots viewed along < 110 > for the (**a**) thermodynamic, (**b**) kinetic, (**d**) thermodynamic modified and (**e**) kinetic modified with re-entrant enhancement Winterbottom constructions λ and ${\text{v}}$ correspond to surface energy and growth velocity, respectively, and γ$_{\text{eff}}$ and ${\text{v}}_{\text{eff}}$ correspond to the effective interface energy and effective growth velocity, respectively. The twin energy/growth velocity is considered to be negligible. TEM images of (c) a single Pt crystal grown on a SrTiO_3_ nanocube, adapted with permission from [46], and (**f**) a five-fold Au Marks decahedron grown on a faceted LnScO_3_ nanoparticle, adapted with permission from [19]. Copyright (2021) American Chemical Society

Nanocrystal applications may require or benefit from support on a substrate, for instance in catalysis where this can improve performance and reduce sintering [46–48]. The thermodynamic Winterbottom construction (Fig. 4), developed in 1967 [18], allows for the determination of crystal shape grown directly on a flat substrate (rather than deposited) by adding an interface with positive or negative adhesion energy ${\gamma }_{A}$. This term is added to the surface free energy ${\gamma }_{i}$ of the facet *i* being replaced by the interface to yield ${\gamma }_{int}$, an effective interfacial energy term:

9 $$\begin{array}{c}{\gamma }_{int}= {\gamma }_{i}+ {\gamma }_{A}.\end{array}$$

This interfacial energy then replaces the surface energy term for that facet in an otherwise standard Wulff approach.

The thermodynamic Winterbottom construction has been used or invoked in multiple contexts, often related to supported catalysts, including the epitaxial growth and resulting orientation of La_1.3_Sr_1.7_Mn_2_O_7_ on LaAlO_3_ [49] and the shape of Pt nanoparticles on SrTiO_3_ [46].

The *modified Winterbottom construction* was recently developed [19] for supported nanocrystals with internal (twin) boundaries such as MTPs. Similarly to the thermodynamic Winterbottom construction, an effective interfacial energy term ${\gamma }_{eff}$ is introduced for the crystal-substrate interface; this replaces the ${\gamma }_{int}$ term for single crystals by allowing different contributions from multiple differently oriented sub-units. As the truncation height of the nanocrystal on the substrate, *h,* varies, so does the relative areal contributions from each crystalline subunit *A*_*j*_ and consequently the interfacial energy. Hence, ${\gamma }_{eff}$ varies with *h*:

10 $$\begin{array}{c}{\gamma }_{eff}\left(h\right)= \sum {\gamma }_{int\left(\widehat{n}\right)}{f}_{j}\left(h\right),\end{array}$$

where ${\gamma }_{int\left(\widehat{n}\right)}$ is the orientation-dependent local interfacial energy between subunit *j* and the substrate and *f*_*j*_ is the proportion of the total interfacial area taken up by each individual subunit. The energy-minimizing shape can thus be determined, as for the single crystal Winterbottom construction by using ${\gamma }_{eff}$ along the interface. The result is a nanocrystal adopting a truncated height and orientation such that the total energy is minimised.

The case of a supported decahedral particle of FCC has been explored in detail [19] and matches well with experimental evidence. Extensions to other twinned shapes and comparison with experimental data should be straightforward as long as one considers that the approach may only yield local, rather than global, minima.

The kinetic variants of both the single crystalline and modified Winterbottom constructions can be implemented by using growth velocities instead of surface energies. While no kinetic enhancement effects (concave surfaces, twin boundaries, etc*.*) have been formally implemented thus far, the *kinetic Winterbottom construction* has been used in several analyses including the growth of GaN islands and nanoparticle superlattices [41, 50–52].

Additional variants of supported crystals have been developed for specialized contexts. The Summertop construction [20] is an extension of the Winterbottom construction for a nanocrystal between two interfaces, while the double-Winterbottom construction [53] allows for deformable substrates with non-zero mobilities.

### The “atomistic” Wulff constructions

What has been called in the literature the atomistic Wulff construction is not in fact a distinct Wulff construction or arguably a mathematical approach at all, but we take the opportunity here to disambiguate its meaning and context. This is not a separate Wulff-related construction in the same sense as the models looked at until now—it is simply an application of the classic thermodynamic Wulff model.

**Fig. 5:**
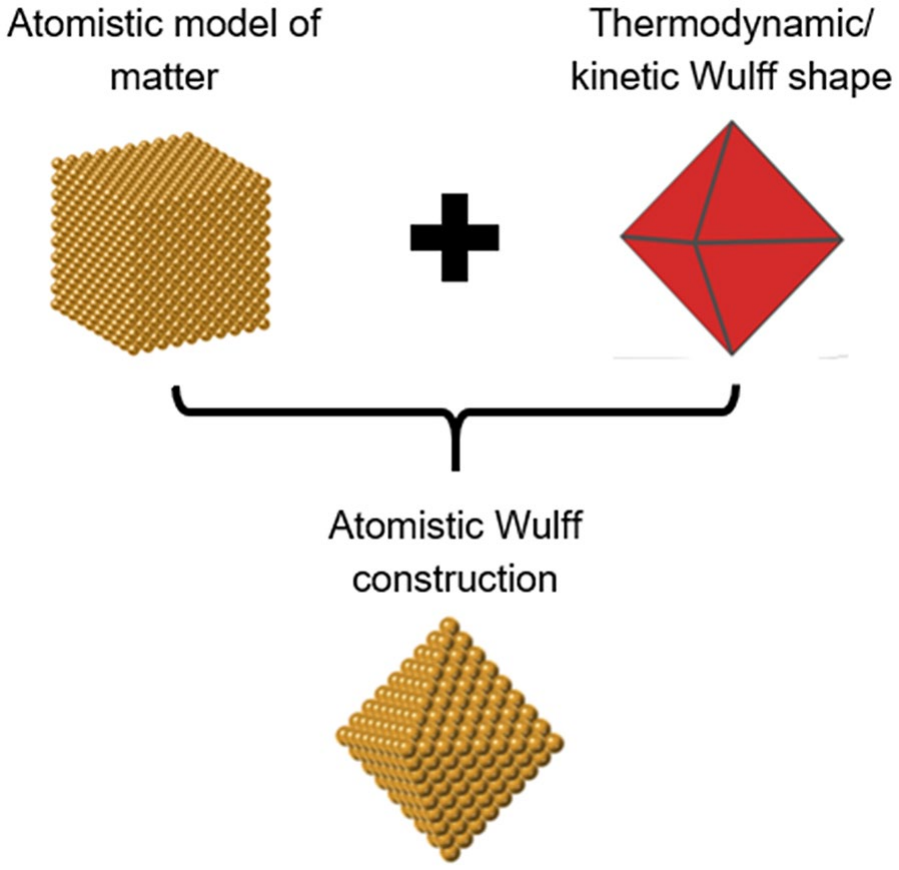
Schematic of the steps required to generate an atomistic Wulff construction, here shown for a Au octahedron. The Wulff shape was rendered in Crystal Creator [32], the atomistic representation was obtained from NanoCrystal [57] and the atomistic illustrations were created in CrystalMaker [58]

The term atomistic Wulff construction came into use in 2005 with the first of a series of papers on modelling equilibrium-shaped Ru-based catalysts used in industrial ammonia synthesis [54–56]. There, Density Functional Theory (DFT) was used to predict the ${\gamma }_{\left(\widehat{n}\right)}$ used in the thermodynamic Wulff construction (Eq. 1). The additional step making this “atomistic” is the filling of the resulting shape by atoms arranged according to the lattice and exposing the surface packing characteristic of each facet (Fig. 5). Thus the atomistic Wulff construction is simply an application of the standard thermodynamic Wulff construction in which the resulting shape is filled with model atoms.

While filling a shape with model atoms is not by itself a Wulff construction, it is useful in a variety of contexts not covered by the continuum result. The resulting atomistic models of nanocrystals have been used for modelling surface adsorption [59–61] and active sites for catalysis [54–56, 62]. Moreover, it could also be useful in the case of very small particles, if they retain the same packing as the bulk. Indeed, considering the need to fit integer numbers of atoms leads to a fixed number of possible nanocrystal sizes with “magic numbers” of atoms. For example, the five smallest icosahedra have 13, 55, 147, 309 and 561 atoms each [63, 64].

### Terminology and ambiguities

While the notion of Wulff construction as a shape-predicting tool is generally well-known in the nanotechnology literature and its increased use is welcome, the existence and specific applicability of the multiple variants discussed above are not always acknowledged correctly. There is not, unfortunately, a single Wulff construction applicable to all cases, and much confusion could be avoided by unambiguously specifying which approach (thermodynamic vs kinetic, modified, supported, etc*.*) is used.

An early source of confusion is Wulff’s 1901 paper [13] itself: it actually describes a shape controlled by the speed of crystal growth, yet remains the foundational paper for the thermodynamic Wulff construction. This seeming contradiction is solved by Wulff’s assumption of a direct relationship between surface energy and growth rate, setting up the framework for a thermodynamic construction. Yet it is no surprise that kinetic and thermodynamic arguments remain muddled in the current literature. Indeed, a significant number of recent papers incorrectly described kinetically controlled nanocrystal shapes as thermodynamic ones [65–67] with others explicitly stating that Wulff constructions can *only* be used to determine thermodynamic equilibrium [68, 69]. The propagation of the incorrect idea of “one Wulff construction” has not exclusively led to confusion regarding kinetics *vs* thermodynamics: the Winterbottom construction has been referred to as “the Wulff construction” [70] or “modified Wulff construction” [71], and the kinetic Wulff construction has been called a “modified Wulff construction” [72]. These names are often not strictly incorrect, but the use of a more precise terminology would clarify which mathematical construct is used and help readers apply them in their own systems.

**Fig. 6:**
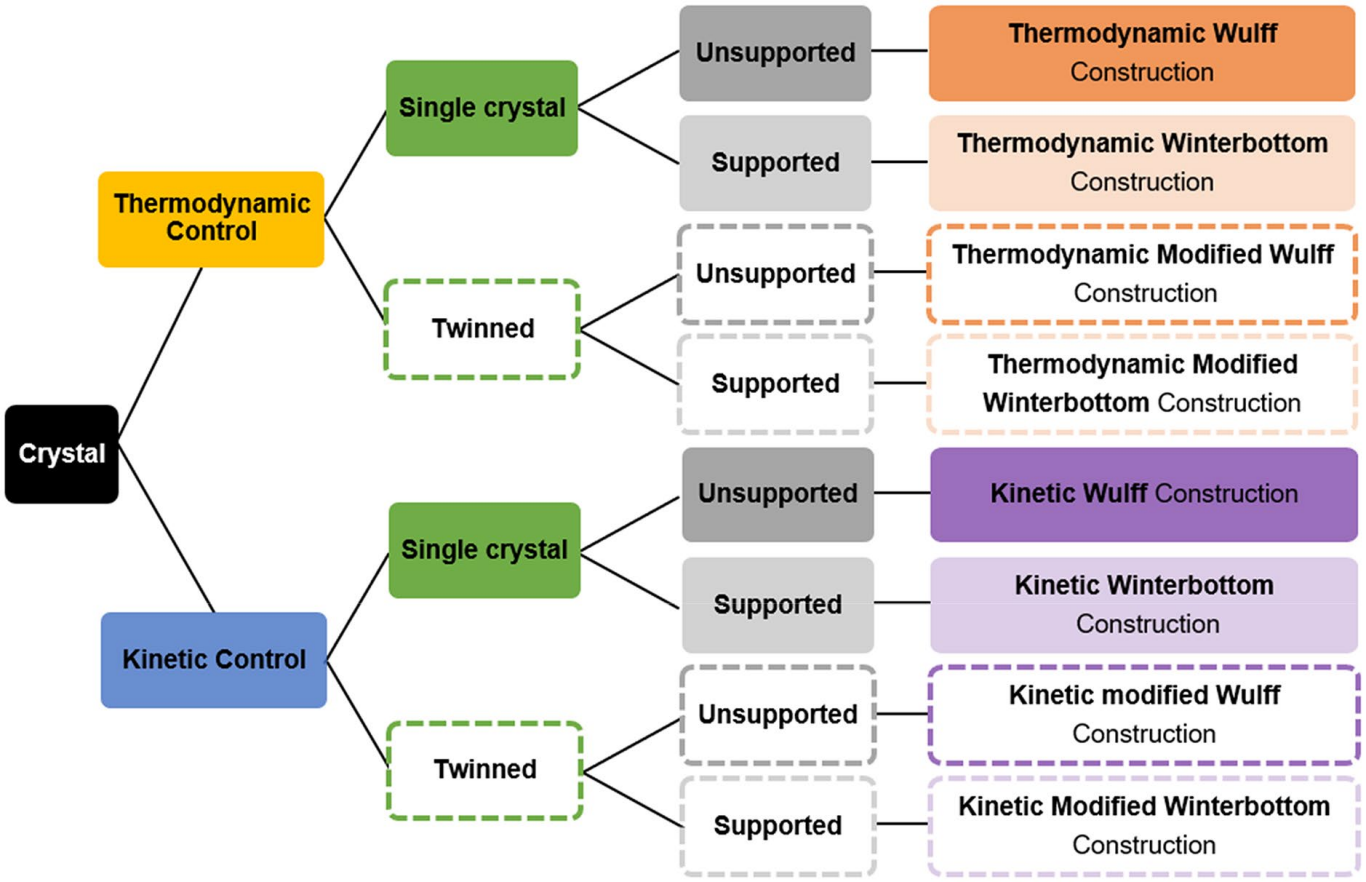
Diagram of the terminology of the various shape constructions based on the considered surface property (energy for thermodynamic, growth velocity for kinetic), crystal structure (single or twinned crystal) and the growth environment (supported or unsupported)

Other names, not incorrect but also perhaps adding to the confusion, exist for the thermodynamic Wulff construction, such as “the equilibrium Wulff construction” [73–76], “the Gibbs-Wulff construction” [77–81], “the original Wulff construction” [82], “the classical Wulff construction” [14, 67, 83–88], and “the traditional Wulff construction” [89]. Lastly, the “atomistic Wulff construction” terminology is not quite correct in the instances where the shape is derived from continuum approaches; alternative naming, or simply no specific name [60, 63] should be used. We direct readers to Fig. 6 for a clear map of the various names of Wulff-related constructions.

## Computational tools for the modelling of crystal shape

Since interest in crystal shape started gaining momentum, initially to model minerals and later nanoparticles, an increasing variety of computational tools have been developed to implement Wulff-like constructions. The list of available tools is quite large and includes both web-based and desktop applications such as Wulffman [90], WinXMorph [91], Wulffmaker [53], BCN-M [92], NanoCrystal [57] and Crystal Creator [93] as well as open source codes such as Wulffpack [94] and SOWOS [95]. These can construct a variety of crystal shapes based on user input that typically involves defining the crystal structure, the exposed facet orientations (denoted $\widehat{n}$ or i) in the form of Miller indices, and their associated surface energies (or growth velocities) ${\gamma }_{i}$. The first step, common to all tools, is to identify the involved facets based on the crystal symmetry. Analytical calculations of the resulting shape involve a convexification process of $\widehat{n}{\gamma }_{(\widehat{n})}$, achieved by means of constructing a dual Wulff shape or by closest vertex identification techniques. A numerical approach, adopted in Crystal Creator [93], has also been reported, where the shape construction is based on the calculation of growth velocities over discretized space. Further, the increasing applicability of Wulff approaches in nanoparticle modelling has prompted an interest in tools that overlay atomic structure to Wulff-derived shapes, such as BCN-M [92] and NanoCrystal [57]. In this Section, we describe the implementation of various Wulff constructions and discuss their advantages and limitations.

### Dual Wulff shape

One approach to solving the Wulff construction problem, implemented in Wulffman [90] and very recently in Wulffpack [94], involves the calculation of the dual Wulff shape followed by a convex hull calculation. These algorithms first calculate the set of planes to be considered based on the crystal structure symmetry, then a shape is constructed based on the principle of duality, *i.e.* that each plane has a dual point and vice versa. Briefly, a set of dual points with vectors $\left(\frac{h}{{h}^{2}+{k}^{2}+{l}^{2}}, \frac{k}{{h}^{2}+{k}^{2}+{l}^{2}}, \frac{l}{{h}^{2}+{k}^{2}+{l}^{2}}\right)$ are calculated for each facet $(h,k,l)$ and a convex hull algorithm is applied to compute the dual Wulff shape, *i.e.* a set of the dual plane normals and vertices. Calculating the dual of that shape yields the vertices and plane normals of the Wulff shape itself.

Wulffman is one of the first user-friendly applications developed to calculate the Wulff shape for crystals of any crystallographic symmetry. The user needs to define, at minimum, the shape’s point group symmetry, selecting either one of the 32 crystal classes, one of the 2 icosahedral point groups or a custom-made point group, the crystallographic vectors and angles, and the desired crystal facets along with their surface energies. Additional input can be provided to simulate dynamic Wulff constructions, to construct partially faceted shapes with smooth connecting regions, or to introduce non-symmetry related facets, a feature that can be exploited to simulate cleavage. Finally, multiple shapes can be created independently in the same window allowing for simulation of shape intersections. The user can define the colour and visibility of the facets and vertices, obtain geometric information such as selected distances, point coordinates and facet areas, while statistics on the number of facets, edges and vertices, as well as total volume and surface area are readily provided. Shapes can be exported as still or animated GIF files. Wulffman is a very versatile tool, however it features quite old graphics and has not been updated since 2002. It is available to download as a binary or C++ source code [96] and can also be run online via nanoHUB [97].

WulffPack [94, 98] is a recently developed open source Python package that, in addition to single crystal shapes, implements the Winterbottom construction, twinned decahedral and icosahedral shapes as well as atomistic overlays. The user creates a python script calling the class of the relevant construction and, for a single crystal, provides the primitive structure type, in order to define any of the crystallographic point groups, the facets and their surface energies. Additionally, the twin energy is required for decahedral and icosahedral shapes, although in reality this is much lower than the surface energies and thus is typically negligible. The interface energy and direction are required for Winterbottom constructions. For the atomistic representation, the total number of atoms is required while the stoichiometry is extracted via the provided primitive structure. The twinned constructions and the atomistic representations are supported only for cubic systems and the primitive structure is defined via classes available in the python atomic simulation environment (ASE) [99]. The computed Wulff shapes can be visualised using matplotlib, a library which allows for a variety of visual customizations, or exported in OBJ (.obj) format, while the atom positions in the atomistic representations can be visualised using the ASE graphical user interface (GUI) or exported as a text file. An available web application powered by Wulffpack [100] offers an excellent and quick visualisation of various shapes, although it is restricted to cubic crystals and does not include the Winterbottom construction.

### Closest vertex technique

Another popular approach to render crystal shapes is that of vertex elimination. It is based on the principle that the projection of any point belonging in the Wulff shape on any facet plane normal must be shorter than the normal vector’s length. Hence, after the equivalent facets are populated based on the (user-defined) crystal symmetry, a gamma plot-like vector normal to the plane and with length equal to the corresponding surface energy is defined for each facet. Possible shape vertices are then calculated by the intersection of sets of three planes. If the projection of a vertex vector onto one of the gamma vectors is longer than the length of the gamma vector then the vertex is out of the shape and is eliminated. The remaining vertices are arranged in order, such that they circumscribe the facets. This approach is typically slower than the dual Wulff shape but additional techniques can be implemented to make the algorithm faster: in Wulffmaker [53], for instance, high energy facets are eliminated by a similar process before computing all the possible vertices.

The Wulffmaker [53] GUI has been developed as a replacement for Wulffman, featuring an upgraded interface and capabilities. Wulffmaker supports single crystal and Winterbottom constructions as well as what is termed a double Winterbottom construction, describing shapes of particles attached to deformable interfaces. The user selects any of the 32 crystallographic symmetries, specifies crystallographic vectors and required angles and can define up to 30 symmetrically distinct facets with their corresponding energies. For the Winterbottom and double Winterbottom constructions additional input on the interface direction and energy is required. For the double Winterbottom construction, a feature unique to Wulffmaker, two separate shapes are created and subsequently their intersection is calculated. For all approaches, the shape is displayed as an interactive 3D object with facet colouring options and information on the relative surface areas. Wulffmaker is distributed [101] either as a Wolfram Mathematica code or as a computable document format (.cdf) file, the later easily used via the free Wolfram.cdf visualizer.

WinXMorph [91, 102] is a Microsoft Windows application, [103] written on Delphi 6, primarily developed to model realistic still or animated representations of crystal shapes. However, in contrast to most tools described here it also allows for modelling a variety of twinning types. The minimum user input, as usual, consists of one of the 32 crystallographic point groups, the crystallographic axes and angles, and the facets to be considered and their associated energies. These can be inserted manually or loaded via a typical crystallographic information file (.cif). In addition, the user can insert a non-symmetry related plane to simulate cleavage, or introduce one of the three twinning types available. Specifically, the user can name the twin plane to simulate contact twins or inversion twins and the rotation axis, rotation angle and boundary plane to model rotation twins. Interestingly, there is no mention of the Wulff construction in the relevant WinXMorph publications or in the extensive help documentation available, despite being obviously related. WinXMorph goes beyond Wulff approaches and offers the option to use the Bravais-Friedel-Donney-Harker (BFDH) model [104–106] to predict crystal morphology, an approach based on the assumption that the size of a facet is inversely proportional to the lattice spacing of that facet (this is also available in the software Mercury CSD 2.0 [107]).

WinXMorph excels at rendering and allows control over the colour, transparency, lighting, and texture of the crystal, independent of any symmetry relations, achieving artistic to realistic visualisations. Calculated shapes can be exported as .stl or. gif files and as virtual reality files in the VRMLV2.0 format; some information is given on the total volume and area of the constructed crystal.

SOWOS [95], which stands for solid of Wulff open source, is a Fortran90 code [108] designed for a Linux environment and developed initially to model self-assembled heteroepitaxial islands. It can model Winterbottom and single crystal constructions, and includes an option for atomistic overlays. The input consists of two text files, one lists the facets and their corresponding energies paired with a multiplicity factor and the other, a path to the first file as well as information for the atomistic simulations. The multiplicity factor defines how many facets whose normal vector forms the same polar angle with the [001] direction need considering. The output information is provided in 20 separate files, including text files (.dat), coordinate files (.xyz), class files (.cls) and vector-based plotter files (.plt) containing, for instance, the volume, surface areas, vertex coordinates, and facet normals. This information can be used to visualise the constructed shape via different software such as gnuplot or COMSOL.

NanoCrystal [57] is a web application [109] developed to provide a simple and quick tool for computing the atomistic representation of single crystals. The user needs to upload a typical crystallographic information file (.cif) containing all relevant symmetry and atomic information, as well as the facets to be considered and their surface energies. The maximum nanoparticle radius is also required and the user can choose between truncating the shape exactly at the Wulff shape boundary or to include only the atoms that form coordination polyhedra with atoms inside the shape. An interactive three dimensional shape of the atomic arrangement is generated on the web and atomic positions can be downloaded in the form of .xyz or .pdb files.

### Growth front isosurface technique

Crystal Creator is a standalone user-friendly GUI capable of both kinetic and thermodynamic, single crystal and twin Wulff constructions [32, 93]. Additionally, it can generate a 3D dipole array for complex shapes and core–shell particles of FCC, BCC and HCP structures, as well as the necessary input files for DDSCAT-based [110] electromagnetic simulations. In this approach, space is discretized in a cubic three-dimensional grid and growth velocities are calculated at each point of the grid by computing the contribution of all the different considered facets. The shape is then defined as an isosurface of growth velocities; the isosurface value is proportional to the distance of the facet from the geometric centre of the particle. This computational approach is key to allowing optional enhancements at re-entrant surfaces, twin boundaries, and disclinations enabling the modelling of a wide range of kinetic shapes that cannot be readily modelled via other software. Surface facets are currently restricted to low indices, namely {100}, {110} and {111} for FCC, {100}, {110}, {111} and {112} for BCC and {0001}, {${10\bar{1}0}$}, {${10\bar{1}1}$} and {${10\bar{1}2}$} for HCP, but expansion to higher order facets is straightforward. All common twin planes are encoded for the construction of singly twinned as well as FCC five-fold twinned structures; these are the (111) for FCC, (112) for BCC and ($10\overline{1 }$x), x = 1, 2, 3 and ($10\overline{1 }y$), y = 1, 2, 3, 4 for HCP.

The user input required in Crystal Creator includes selecting one of the three supported crystal structures, setting the surface energies/growth velocities of low index facets, choosing a twinning type and twin plane (or none), setting an optional shell thickness and adjusting some computational parameters such as the grid size. The three dimensional shape is then displayed and can be saved as an image (.png, .tif, .jpeg, etc.) or Matlab figure (.fig) while the coordinates of the grid points in the shape are exported in a text file. The format of this file matches the required DDSCAT input file format; the second file needed for DDSCAT can be interactively produced within Crystal Creator.

The above list of Wulff construction tools is not exhaustive and one can find additional tools. BCN-M [92], which stands for bulk cut nanoparticle model, is a computational tool available as an app and web tool [111] that generates Wulff-like models for binary materials with controlled stoichiometry. Focusing on the atomistic representation, it offers surface termination options that can accommodate stoichiometric nanoparticles, eliminate singly coordinated atoms or feature metal, non-metal, H or OH termination atoms to compensate any facet polarity. KrystalShaper [112] is another tool, built in Java, that models single crystals of any of the 32 crystallographic classes and the 2 icosahedral symmetries and provides functionalities such as Hauy Block [113] and BFDH models, animated gifs, foldable nets, anaglyphs and stereographic projections. Other related tools include the Matlab-based SERPEnT [114], that models the evolution of crystal and surface energy shapes over time, a concept relatable to crystal growth kinetics. Additionally, a Matlab GUI exists to study twin boundaries in HCP crystals [115, 116].

In addition to the tools developed explicitly to model crystal shape, a few large computational packages also include some Wulff-related features, such as VESTA 3, [117] Crystal14, [118] MP-interfaces, [119] Mercury CSD 2.0 [107] and ASE [99]. A draw to these tools is the ability to perform ab initio or other calculations to estimate the surface energies for different conditions, before implementing a Wulff construction. For instance MP [119] (Materials Project) is a Python package developed to study interfacial systems; it offers Wulff construction capabilities using surface energies calculated by taking into account parameters such as ligands on nanoparticle surfaces or lattice mismatch for nanoparticle-substrate interactions.

### Capabilities beyond single crystals: winterbottom and twins

It is worth noting that the Winterbottom construction, explicitly mentioned only in Wulffmaker and Wulffpack, can essentially be modelled by any tool that allows for the addition of a single non-symmetry related facet such as in Wulffman, WinXMorph and SOWOS. The surface energy attributed to the extra facet is the interface energy. Similarly, it is in principle possible to model twinned crystals without the dedicated functions available in Crystal Creator or WinXMoprh: one needs to edit the available codes, as has been reported for BCC twin shapes [120] using Wulffmaker, or input simultaneously multiple modelled single crystals, such as in Wulffman. Of course, this requires the user to be aware of the specific geometric characteristics of the twinned Wulff shape. Lastly, the modified Winterbottom construction is not readily available in any tool at the moment.

### Software selection

The powerful three dimensional visualisations of the various Wulff construction tools can provide an insight into experimentally observed shapes, construct models based on theoretical data, supply the required shape model to further analyse crystal properties, or serve as an aid to understand how crystal symmetry is reflected in the crystal shape. Ultimately, the choice of tool depends on the required construction type (single crystal, twinned, Winterbottom, double Winterbottom), the material’s crystal structure, the preferred tool interface (application or source code) and the purpose of the construction. For instance, if twinning is required one would use Crystal Creator [93] to allow for kinetic enhancements or WinXMorph [91] if a realistic visualisation is required. If additional analysis is required via other software one will consider the compatibility of the tool with that particular software, for example Crystal Creator [93] for DDSCAT, or opt for the more generalised computational packages such as VESTA 3 [117] or MP-interfaces [119]. Web applications generally feature restricted capabilities but can provide a quick visualisation of the crystal shape as well as some output files without any software installation. A summary of the capabilities offered in the main Wulff-modelling software reviewed here is reported in Table 1.

**Table 1:** Summary of the main Wulff-modelling software and their shape construction capabilities

Computational approach	Tool	Wulff	Winterbottom	Modified Wulff		Continuum model	Atomistic model	Stoichiometry for binary systems	Programme language
				Twin	Enhancement				
Dual shape	Wulffman	√	√	×	×	√	×	–	C+ + (1998)
	WulffPack	√	√	√ only Dh & Ih	×	√	√	√some systems	Python (2020)
Closest vertex Technique	Wulffmaker	√	×	×	×	√	×	–	Wolfram Mathematica (2012)
	WinXMorph	√	×	√	×	√	√	×	Delphi 6 (2005)
	SOWOS	√	√	×	×	√	√	√some systems	Fortran90 (2013)
	NanoCrystal	√	×	×	×	×	√	√some systems	C+ + , Matlab, PHP (2018)
Growth front Isosurface	Crystal Creator	√	×	√ not Ih	√	√	×	–	Matlab (2019)

## Bridging shapes and computational results

### Forward modelling

**Fig. 7:**
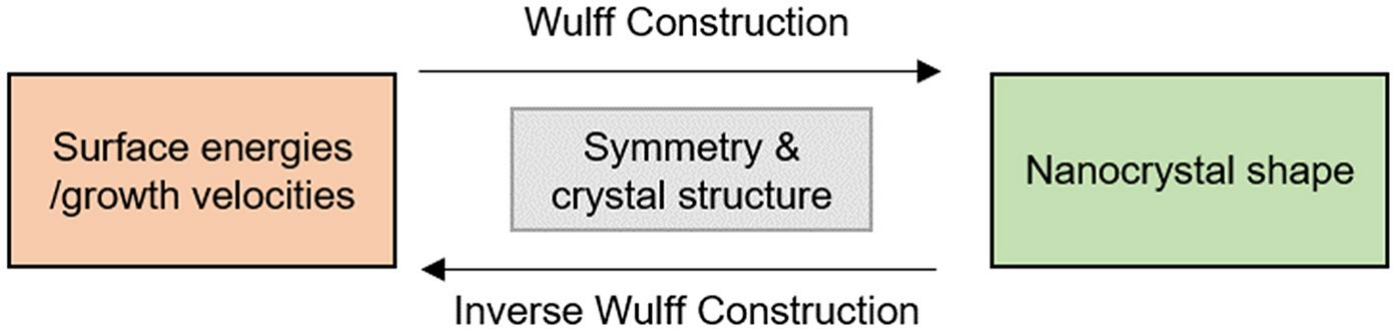
Flow chart illustrating the forward and inverse Wulff construction modelling

The Wulff construction and its various implementations are most commonly used to predict nanoparticle shape based on available knowledge of the exposed facets and the corresponding surface energies as depicted schematically in Fig. 7. Surface energies can be calculated experimentally or computationally, however, both approaches are challenging. Experimental conditions at the interface level are hard to control while computational techniques struggle to model all the phenomena involved without being power- and time-demanding. This often results in inconsistencies on the reported absolute surface energy values for various systems [121, 122]. Fortunately, only the relative values are important for the Wulff construction, so the effect of any errors is minimized.

Surface energies can be obtained numerically from first principles calculations (e.g. DFT, Møller–Plesset perturbation theory [123]), semi-empirical models such as the (modified) embedded atom method (EAM) [124, 125] and the equivalent crystal theory (ECT) [126], or other techniques including the BFDH law and the Hartman-Perdock model [127]. Data is available for low index facets for a variety of metals [121, 128–131] as well as some intermetallics [68]. It is worth highlighting an extensive database [132] incorporated in the interactive Crystalium web app [133] which includes surface energies, often up to miller index 3, of 74 metal and non-metal elements, along with the corresponding single crystal Wulff shape where applicable. These computed surface energies can be used to model single crystals in the thermodynamic regime. Advances in computational tools also allow for the calculation of surface energies for complicated systems such as when ligands [55, 128] or interfaces [119] are present; these can be used to appreciate growth velocities and therefore model shapes in the kinetic Wulff construction. Twin plane energies [134, 135], albeit negligibly small with little influence on the resulting shape, can give an insight into the most probable twin planes allowing for the prediction of twinned crystal shapes [31].

Experimental approaches on surface energy measurements are scarcely reported and are restricted to very few systems. Examples involve cleavage methods [136], calculations from internal free enthalpies of atomization [137], estimation of average solid-vapour surface energies from liquid-surface tension data [138] and more recently a new sessile drop accelerometry technique, exploiting microgravity effects, so far tested only on polymer solids [139]. Additionally, the Wulff construction itself can be exploited to obtain relative surface energy values from experimentally observed shapes as explained in the next section.

Finally, morphing from one shape to another can help instruct the necessary changes in a synthesis to obtain the desired shape. For example, Wulff shape models show that a {100}-faceted FCC cube will shift into a cuboctahedron, expressing both {100} and {111} facets, and eventually into a {111}-bound octahedron, as the {100} to {111} growth velocity ratio increases. Therefore, if starting with a synthesis of cubes one needs to inhibit {111} growth to generate octahedral nanocrystals.

### “Inverse” Wulff construction

The phrase inverse Wulff construction [140, 141] or reverse Wulff construction [142, 143] refers to the use of experimentally determined equilibrium shapes to obtain the ratio of facet-dependent surface energies, as shown in Fig. 7. This approach is ubiquitous in the literature: new nanocrystal shapes are almost always accompanied by some inference of the effect of the growth medium (e.g. capping ligand) on the relative “stability” of the different crystallographic facets. There is often no need for detailed analysis beyond the comparison of shapes obtained in different conditions, and valid qualitative conclusions can be readily obtained and applied to other reactions. A key limitation of this approach is that the results are often kinetic rather than thermodynamic, as many of the additives actually control growth rates rather than thermodynamic properties. It can be difficult to assess whether a reaction is anywhere near thermodynamic, restricting the ability to extract reliable measurements of the thermodynamic properties of the facets. Nevertheless, even when kinetics are at play, the relative growth velocities obtained and their comparison in different conditions can help track the outcome of a synthesis as a function of additives and guide future shape control efforts.

Mathematically, the thermodynamic Wulff construction involves the determination of facet surface areas ${A}_{i}$ by the minimisation of the total surface energy of the particle ${E}_{s}$ where facet-dependent surface energies ${\gamma }_{i}$ are known. In contrast, the inverse Wulff construction involves the determination of surface energies ${\gamma }_{i}$ by the minimisation of the total surface energy of the particle ${E}_{s}$ where facet surface areas ${A}_{i}$ are obtained experimentally:

11 $$\begin{array}{c}{E}_{s}=\sum_{i}{A}_{i}{\gamma }_{i}\end{array}.$$

In practice, measuring surface areas can be challenging, and often determining ${h}_{i}$ is more straightforward. For simple structures such as the continuum of shapes between octahedra and cubes, for instance in Cu_2_O, [144] a growth rate ratio can be easily obtained from these measurements performed on electron micrographs of the nanoparticles. For more complex and twinned structures, iterative comparison with predicted shapes, often along multiple viewing directions [26, 31] is the simplest approach, highlighting the need for platforms capable of producing a variety of twinned shapes, as described in Sect. 3.

Given one surface energy value, often determined by DFT, others can be deduced using the ratios. Such quantitative results can be used as inputs for other thermodynamic material models [14], while information on either surface energies or growth velocities can guide new ligand design and synthetic approaches.

## Conclusion

As interest in synthesising (nano)crystals increased over the past decades, a plethora of mathematically distinct Wulff-based approaches have been developed to describe and predict their shapes. These models were reviewed and include thermodynamic and kinetic approaches, single crystal and twin shapes, as well as suspended and supported systems, as summarized in Fig. 6. Misconceptions about and unawareness of the nuances of these many models are common in the nanotechnology literature, leading to confusing statements and misclassified results. This review provides the background that scientists need to communicate clearly about crystal shape models.

All except the most recent construction, the modified Winterbottom, have been implemented in one or more openly or commercially available software packages, making crystal shape modelling quick and user-friendly. We described such approaches alongside their capabilities and limitations, thus providing a guide for their use in a variety of contexts.

Looking to the future, the increasingly complex crystal shapes and compositions developed to fulfil the exciting promises of nanotechnology will require increasingly agile descriptive and predictive tools. Wulff-related constructions are capable of supporting such scientific advances owing to their many different modifications accumulated over a century. At present, all of the required mathematical foundations are available, and only further, more streamlined and complete implementations are to be developed and hopefully widely adopted.

## Supplementary Information

**Additional file 1**:** Fig. S1.** Total number of publications featuring the words “nanoparticle” (grey) and both the words “nanoparticle” and “shape” (blue) in their title or abstract.

## Data Availability

Data available upon request.
