# Supplementary material for: Approaches to modelling the shape of nanocrystals
Source: Nano Converg. 2021 Sep 9;8:26. doi: 10.1186/s40580-021-00275-6 (PMC8429535; doi:10.1186/s40580-021-00275-6)
Supplement: Supplementary file 1 — Additional file 1: Fig. S1. Total number of publications featuring the words “nanoparticle” (grey) and both the words “nanoparticle” and “shape” (blue) in their title or abstract. [file 40580_2021_275_MOESM1_ESM.docx]

**Additional file 1**

**Approaches to Modelling the Shape of Nanocrystals**

Christina Boukouvala,^1,2†^ Joshua Daniel,^1†^ Emilie Ringe^1,2*^

^1^ Department of Materials Science and Metallurgy, University of Cambridge, Cambridge CB3 0FS, United Kingdom

^2^  Department of Earth Sciences, University of Cambridge, Cambridge CB2 3EQ, United Kingdom

^†^ These authors contributed equally

* Corresponding author: Emilie Ringe

Tel.: + 44 (0) 1223 334300, E-mail address: [er407@cam.ac.uk](mailto:er407@cam.ac.uk)


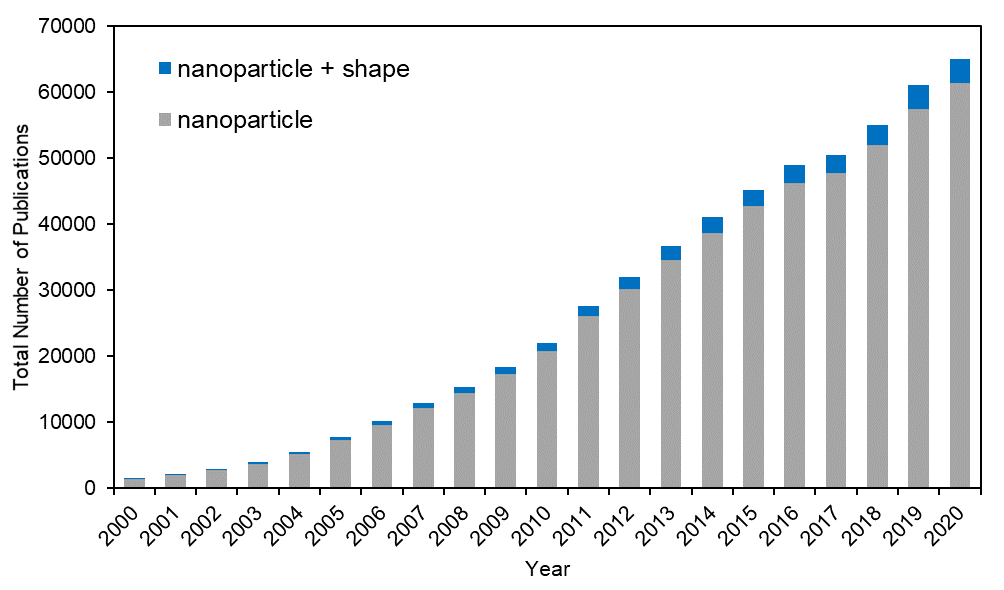


**Fig. S1.** Total number of publications featuring the words “nanoparticle” (grey) and both the words “nanoparticle” and “shape” (blue) in their title or abstract. Data obtained from https://app.dimensions.ai/discover/publication.
